# *PlasmoView*: A Web-based Resource to Visualise Global *Plasmodium falciparum* Genomic Variation

**DOI:** 10.1093/infdis/jit812

**Published:** 2013-12-12

**Authors:** Mark D. Preston, Samuel A. Assefa, Harold Ocholla, Colin J. Sutherland, Steffen Borrmann, Alexis Nzila, Pascal Michon, Tran Tinh Hien, Teun Bousema, Christopher J. Drakeley, Issaka Zongo, Jean-Bosco Ouédraogo, Abdoulaye A. Djimde, Ogobara K. Doumbo, Francois Nosten, Rick M. Fairhurst, David J. Conway, Cally Roper, Taane G. Clark

**Affiliations:** 1 Department of Pathogen Molecular Biology, London School of Hygiene and Tropical Medicine, Keppel Street, London WC1E 7HT, United Kingdom; 2 Malawi-Liverpool-Wellcome Trust Clinical Research Programme, Blantyre 3, Malawi; 3 Liverpool School of Tropical Medicine, Liverpool L3 5QA, United Kingdom; 4 KEMRI-Wellcome Trust Research Programme, Kilifi, Kenya; 5 Department of Infectious Diseases, Heidelberg University School of Medicine, Heidelberg 69120, Germany; 6 KEMRI-Wellcome Trust Research Programme, Kilifi, Kenya; 7 King Fahd University of Petroleum and Minerals, PO Box 468, Dhahran 31262, Kingdom of Saudi Arabia; 8 Papua New Guinea Institute of Medical Research, PO Box 483, Madang, Papua New Guinea; 9 Oxford University Clinical Research Unit, Wellcome Trust Major Overseas Program, Hospital for Tropical Diseases, District 5, Ho Chi Minh City, Vietnam; 10 Institut de Recherche en Sciences de la Sant, Bobo–Dioulasso, Burkina Faso; 11 Institut de Recherche en Sciences de la Sant, BP 545, Bobo-Dioulasso 01, Burkina Faso; 12 Malaria Research and Training Centre, Faculty of Medicine, Pharmacy and Dentistry, University of Bamako, Bamako, Mali; 13 Wellcome Trust Sanger Institute, Hinxton CB10 1SA, United Kingdom; 14 Malaria Research and Training Centre, Faculty of Medicine, Pharmacy and Dentistry, University of Bamako, Bamako, Mali; 15 Mahidol-Oxford Tropical Medicine Research Unit, Bangkok 10400, Thailand; 16 Centre for Tropical Medicine, University of Oxford, Oxford OX3 7LJ, United Kingdom; 17 Shoklo Malaria Research Unit, Mae Sot TAK 63110, Thailand; 18 Laboratory of Malaria and Vector Research, National Institute of Allergy and Infectious Diseases, National Institutes of Health, Bethesda, Maryland 20892, USA

**Keywords:** *Plasmodium falciparum*, malaria, genomics, drug resistance, vaccine targets, visualization

## Abstract

Malaria is a global public health challenge, with drug resistance a major barrier to disease control and elimination. To meet the urgent need for better treatments and vaccines, a deeper knowledge of *Plasmodium* biology and malaria epidemiology is required. An improved understanding of the genomic variation of malaria parasites, especially the most virulent *Plasmodium falciparum* (*Pf*) species, has the potential to yield new insights in these areas. High-throughput sequencing and genotyping is generating large amounts of genomic data across multiple parasite populations. The resulting ability to identify informative variants, particularly single-nucleotide polymorphisms (SNPs), will lead to the discovery of intra- and inter-population differences and thus enable the development of genetic barcodes for diagnostic assays and clinical studies. Knowledge of genetic variability underlying drug resistance and other differential phenotypes will also facilitate the identification of novel mutations and contribute to surveillance and stratified medicine applications. The *PlasmoView* interactive web-browsing tool enables the research community to visualise genomic variation and annotation (eg, biological function) in a geographic setting. The first release contains over 600 000 high-quality SNPs in 631 *Pf* isolates from laboratory strains and four malaria-endemic regions (West Africa, East Africa, Southeast Asia and Oceania).

Malaria parasites cause disease in approximately 650 million people and *Plasmodium falciparum* (*Pf*) in particular kills up to 1 million people each year [1]. Antimalarial drug resistance is a major public health problem that hinders disease control and elimination efforts [2]. *Pf* parasites from almost all malaria-endemic countries show modest levels of drug resistance, especially to chloroquine [3]. Recent evidence indicates that *Pf* parasites in Cambodia and Thailand are developing resistance to artemisinin – currently the most effective anti-malaria intervention [4–7]. An improved understanding of *Pf* genetics has provided new insights into the molecular mechanisms of drug resistance [8–10] and may ultimately lead to new treatments and reduce the global disease burden [11].

High-throughput sequencing technologies and large-scale genotyping chips are generating genome-wide *Pf* data on an unprecedented scale. This means that it is now possible to densely map genomic variation (eg, single-nucleotide polymorphisms, SNPs) and assess global diversity. Knowledge of the diversity of variants across populations will enable the biological interrogation of novel mutations and identification of candidate vaccines. Other potential applications include SNP-based assays to barcode parasites over time and space for epidemiological, diagnostic and clinical studies. However there are several roadblocks to the translation of genomic variation into useful public health tools. These include difficulties in obtaining robust phenotypic data (eg, drug resistance and mosquito transmission) on samples and the lack of web-based informatics tools to access robust genomic data, which are needed to launch further experiments and translational activities.

Whilst the *plasmodDB* [12] and *genedb* [13] web-browsers provide genomic annotation and support the investigation of SNPs in individual parasite strains, there is a need for additional information (eg, allele frequencies and population statistics) across the growing collection of publicly available sequences and genotypes from clinical *Pf* isolates*.* We are making available to the malaria community the *PlasmoView* web-browser (http://pathogenseq.lshtm.ac.uk/plasmoview) to facilitate the investigation of genome-wide polymorphisms in parasites from different malaria-endemic regions.

## RESULTS

### SNP Data

We aligned raw sequence and genotyping data (to the 3D7 reference genome, version 3, 80.9% AT, 23 Mb, 14 chromosomes) from 971 *Pf* samples from four regions: West Africa (WAF, Burkina Faso, Gambia, Ghana, Mali and Senegal), East Africa (EAF, Kenya and Malawi), Southeast Asia (SEA, Cambodia, Thailand and Vietnam) and Oceania (OCE, Papua New Guinea, Supplementary Table S1). After quality control procedures, 631 samples (8 laboratory/reference, 367 WAF, 88 EAF, 151 SEA and 17 OCE) were retained. The alignments and high coverage (>10-fold) enabled the identification of 593 579 (2.6% of all nucleotides) high-quality SNPs across the nuclear genome (51% exonic, 33% non-synonymous, Supplementary Figure 1*A*). These SNPs include the 86 158 SNPs identified in 227 samples [14]. Seventy-three per cent (434 290) of SNPs are rare (minor allele frequency, MAF < 1%) and 4% (29 036) are common (MAF > 10%, see Supplementary Figure 1*B*).

The linkage disequilibrium between non-rare markers reveals decay with physical genetic distance (Supplementary Figure 1*C*). This decay is higher in the African regions (WAF and EAF) than in SEA or OCE. As expected, a principal component analysis reveals that the SNP data differentiate the samples by geographic region (Supplementary Figure 1*D*) and population (not shown). To identify the polymorphisms driving the population differentiation, we applied a SNP-wise *F_ST_* approach where values between 0 and 1, higher values imply more differentiation [15]. The application across regions identifies 467 SNPs with *F_ST_* > 0.1 indicating a high level of regional differentiation. As expected, the *F_ST_* values of intra-region comparisons are substantially lower (% SNPs with *F_ST_* > 0.1: WAF 0.2%, EAF 1.6%, SEA 6.6% and OCE n/a) than inter-region comparisons (% SNPs with *F_ST_* > 0.1: 27.9%) in common SNPs (MAF > 15%).

### PlasmoView

The web-based *PlasmoView* tool presents genome-wide variation and geographical information on *Pf*. The implementation contains a first release of over 370 million data points (approximately 600 k SNPs in over 600 samples described above). *PlasmoView* provides real-time visualisation and summary statistics and is a timely tool for the high-level interrogation of large genomic datasets. *Pf* data are presented in two views: (a) the matrix view provides a colour-coded indication of mutation locations ordered by genetic and geographical location; and (b) the map view shows a global view of SNP prevalence by country. The matrix view includes graphical and real-time textual SNP-by-SNP genomic annotation (reference alleles, subtelomeric regions, gene regions, amino acid changes and genomic uniqueness) as well as MAF and *F_ST_* graphs plotted for the selected data. Both matrix and map views are interactive enabling researchers to easily navigate the data via the menu at the top of the application or using mouse buttons. The *PlasmoView* launch page includes a facility to search by common gene name (eg, *RH5* or *CRT*), previous id (*PFD1145c* or *MAL7P1.27*) and the latest gene nomenclature (*PF3D7_0424100* or *PF3D7_0709000*). The tool is scalable for the significant increase in data that is anticipated over the next few years. In the next sections we demonstrate the utility of *PlasmoView* using specific loci in the *Pf* genome.

**Figure 1.**
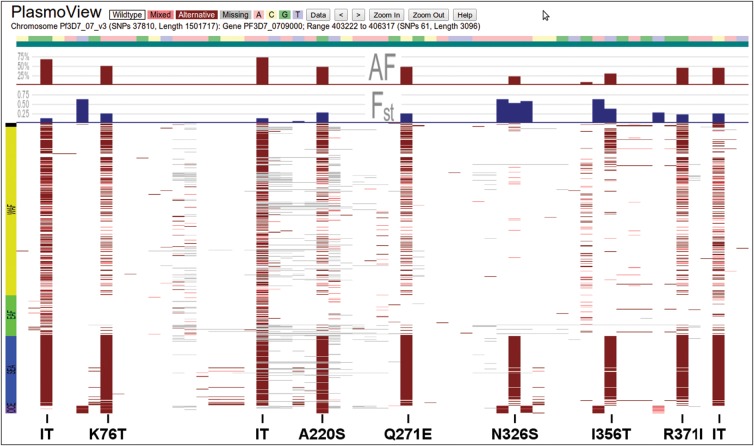
Global mutations in the *Pf* chloroquine resistance transporter gene (*PfCRT*). The 61 SNPs (18 non-synonymous) identified in *PfCRT* (Chromosome 7, *PF3D7_0709000*) are shown. Some markers (eg, Q271E) show evidence that they have gone to fixation in SEA (blue on the left axis) and are common throughout the rest of the world (WAF: green; EAF: yellow; OCE: purple on the left axis). Six known drug resistance markers are shown, as well as 3 common SNPs located in intronic regions (IT).

**Figure 2.**
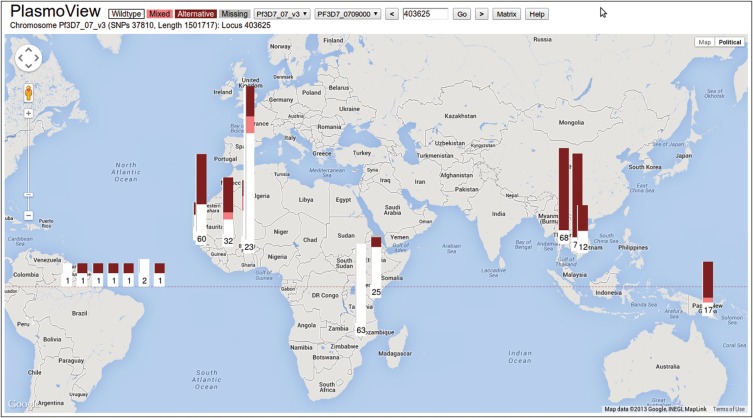
Global distribution of the resistance-conferring *PfCRT* mutation K76T. The global prevalence of the K76T mutation (Chromosome 7, position 403 615) can be seen with the mutation fixed or close to fixed in most countries. Parasites may have reverted back to the wildtype allele, due to the complete withdrawal of chloroquine from some countries (eg, in East Africa, yellow on the left axis). The LAB *Pf* strains (laboratory-adapted and imported *Pf* strains) are located over the South Atlantic. Information on each bar chart is available by holding the cursor over it.

Drug resistance in *Pf* is commonly due to SNPs in genes involved in the biological pathways that antimalarials target. The matrix view in *PlasmoView* is ideally suited to display all observed mutations on a gene-by-gene basis. Figure 1 shows the mutations for each sample in *PfCRT* (*PF3D7_0709000*), a gene on chromosome 7 associated with chloroquine resistance [16]. Sixty-one SNPs (18 non-synonymous) have been detected in this gene, 5 of which are known to be involved in drug resistance. The high regional differentiation is measured by the SNP-wise *F_ST_* (maximum 0.63, see blue histogram track in Figure 1). In *PlasmoView* the geographic spread of any mutation is further visualised in the map view, which displays SNP frequencies by geographical location. For example, Figure 2 shows the frequency of mutations observed across samples from each country at chromosome 7, position 403 615 (the *PfCRT* K76T amino acid mutation, [17, 18]). *PlasmoView* enables the easy identification of mutation sites within each gene, estimate their allele frequencies and investigate their geographical context, via the matrix view, the AF and *F_ST_* graphs and the map view, respectively. The matrix views for three other genes (*PfDHFR*, *PfDHPS* and *PfMDR1*) involved in drug resistance are presented in Supplementary Figures 2*A*–*C* and also provided as part of the set of pre-loaded examples of interest for the web-browser.

**Figure 3.**
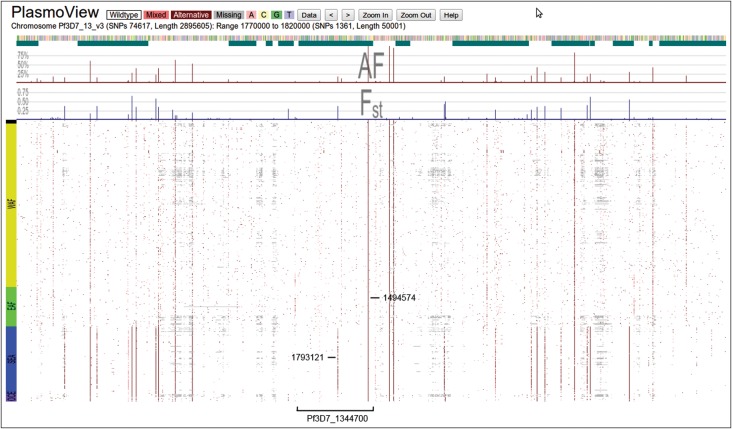
*Pf* chromosome 13, 1770 kb to 1820 kb. Cheeseman et al [8] described this region as being potentially important to the evolution of artemisinin-resistant *Pf* parasites in Southeast Asia. Of the 1355 SNPs in this 50-kb region, more than 20 exhibit high regional specificity (predominantly in SEA with high *F_ST_* (>0.1) and low MAF (<30%)) and at least 10 may have spread or are currently spreading to other regions (high MAF and low *F_ST_*). Gene *Pf3D7_1344700* (see Supplementary Figure 2*D*) contains an example of both: a SNP with high regional specificity (position 1 793 121, labelled as SNP 1 in [8], Supplementary Figure 2*E*) and one moving towards global fixation (position 1 794 574).

Whilst a number of SNPs within drug resistance loci have been well characterised, other more recent and important regions may require investigation. For example, a major region of chromosome 13 (1.73 Mb to 1.82 Mb) in the *Pf* genome was identified to be undergoing a selective sweep due to artemisinin resistance in Southeast Asia [8]. It is possible to visualise the SNP variations in this region for all 631 global parasite isolates and laboratory-adapted parasite strains. The SNP density is very high in this region (1355 SNPs in 50 kb, 1.77 Mb to 1.82 Mb, Figure 3), so we use the SNP-by-SNP information to direct further investigation. There are interesting SNPs progressing to fixation (high MAF and low *F_ST_*) and those showing large geographical differences (high *F_ST_*). For example, the SNP at position 1 793 121 in *Pf3D7_1344700* (Supplementary Figures 2*D* and *E*, MAF 17%, *F_ST_* 0.38) has been identified as having the highest association with parasite clearance rate [[8], denoted SNP 1]. *PlasmoView* shows 46 SNPs in this region whose *F_ST_* is greater than 0.1 including 14 non-synonymous SNPs and one synonymous in *PF3D7_1343800* (22 785 bp, 591 SNPs) and four non-synonymous SNPs and one synonymous SNP in *PF3D7_1344300* (4768 bp, 132 SNPs).

**Figure 4.**
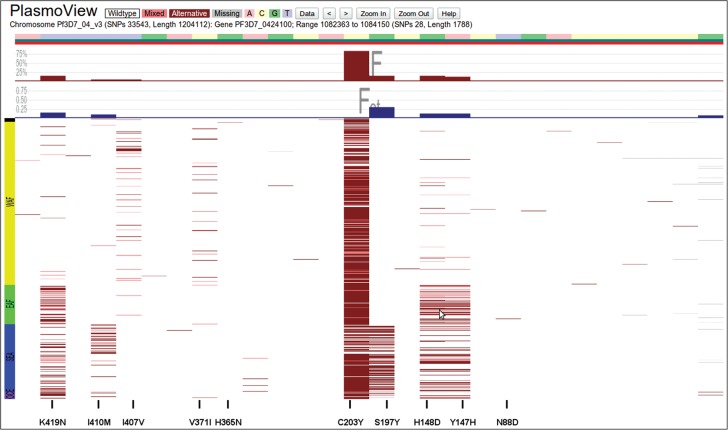
*PfRH5* vaccine candidate in chromosome 4. The *Pf* Reticulocyte Binding Protein Homologue 5 (*PfRH5*, *PF3D7_0424100*) gene is considered a promising vaccine candidate, as it seems to be essential for blood-stage parasite invasion of red blood cells [20]. The locus contains 28 SNPs, including 5 non-synonymous mutations with MAF >10% (I410M, S197Y, H148D, Y147H [19] and K419N [20]).

The lack of an effective licensed vaccine remains one of the most significant gaps in the arsenal to control and eliminate *Pf* malaria [19]. The *Pf* Reticulocyte Binding Protein Homologue 5 (encoded by the *PfRH5* gene, *PF3D7_0424100*) is considered a promising candidate antigen, as it seems to be essential for the invasion of multiple laboratory-adapted *Pf* lines and clinical *Pf* isolates into red blood cells [19]. High genetic variation in target genes can pose difficulties in the design of a vaccine that covers the full range of diversity across populations. The diversity observed for *PfRH5* across 227 clinical Pf isolates was low, with only 5 non-synonymous SNPs (out of 12) reaching a frequency greater than 10% in at least 1 population [19]. Using *PlasmoView* to analyze more than double the number of samples and 5 additional populations, we identify 22 non-synonymous SNPs (out of 28) and confirm the locus has low diversity (Figure 4) with only 5 SNPs exhibiting a frequency greater than 10%.

**Figure 5.**
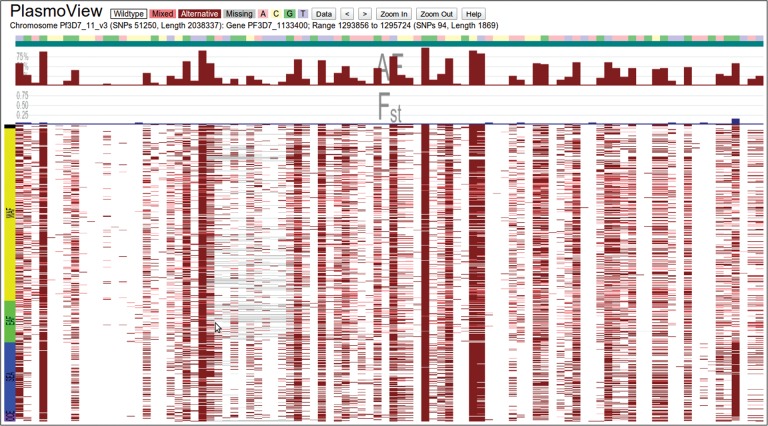
*AMA1* vaccine candidate in chromosome 11. The apical membrane antigen 1 gene (*AMA1*, Chromosome 11, *PF3D7_1133400*) has long been recognised as a vaccine candidate and is currently being evaluated in clinical trials [22]. The high number of SNPs with intermediate MAF and low *F_ST_* indicate that this locus is under balancing selection.

Detecting balancing selection is one method to identify signatures of acquired immunity and therefore potential targets for vaccines. As immunity to the commonest alleles rises in malaria-endemic areas, parasites expressing rarer alleles experience a selective advantage. This process maintains a balance of alleles in the population, with neither the common alleles moving to fixation nor the rare alleles moving to extinction. When multiple alleles are maintained within populations and none of them achieves fixation, balancing selection forces are believed to be present. In *PlasmoView*, loci under balancing selection are readily be visualised as SNPs with intermediate MAF (10% to 40%) and low *F_ST_* (indicating little population differentiation). These signatures are shown in vaccine targets previously identified using methods to detect balancing selection, including the *MSP3.8* (merozoite surface protein 3.8, *PF3D7_1036300*, Supplementary Figure 2*F*) and *AMA1* (apical membrane antigen 1, *PF3D7_1133400*) genes [11, 20, 21]. The malaria vaccine FMP2.1/AS02_A_ is a recombinant protein (FMP2.1) based on *AMA1* and has been tested in clinical trials [22], see Figure 5.

**Figure 6.**
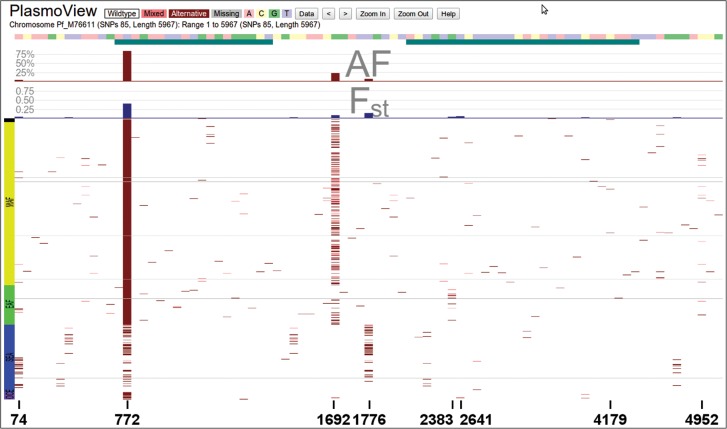
Mitochondrial genome. The *Pf* mitochondrion is a small, uniparentally inherited organelle and its DNA (6 kb) is used for investigating *Pf* evolution and migration [23, 24, 26]. Six SNPs exhibit population differentiation (*F_ST_* > 0.05; *mt74*, *mt772*, *mt1692*, *mt1776*, *mt2383* and *mt2641*) including three common alleles (MAF > 5%; *mt772*, *mt1692* and *mt1776*) used to support an African origin for the species [24, 26].

The *Pf* mitochondrial genome (*Pf_M76611*, 6 kb, 3 genes (*Cox3, Cox1, CytB*), GC content 31.6%) is uniparentally inherited and does not undergo recombination. Therefore mitochondrial DNA (*mt*) sequence divergence and variation data have been used to study the evolutionary history and migration of *Pf* [23–25], particularly gene flow out of Africa. Analyses of the global patterns of *mt* sequence variation have revealed geographic differentiation [24–26]. *PlasmoView* shows 85 SNPs, including the three SNPs with MAF greater than 5% and *F_ST_* in excess of 0.05 (*mt772, mt1692 and mt1776*, see Figure 6), which have previously been utilised in diversity studies [23, 24, 26]. We do not observe a mutation at amino acid position 268 in *CytB (cytochrome B, cob, MAL_MITO_3:4293)* that had been correlated to *atovaquone* resistance following treatment failure [25].

## DISCUSSION

The continued public health burden of malaria and emergence of drug resistance requires the development of new treatments, vaccines and control measures. These efforts are likely to benefit from a deeper understanding of *Pf* biology and malaria epidemiology, in part deriving from analysis of parasite genetic variation. New genomic approaches, using massively parallelisable sequencing and genotyping technologies, are generating vast amounts of *Pf* genetic data. However, to fully achieve their scientific potential, including the initiation of further experiments and translational activities, web-based informatics tools are needed to help researchers to access this genomic information. The *PlasmoView* web-browser condenses and summarises SNP information derived from these technologies into a shared and interpretable visual form. The first release contains nearly 600 k SNPs in over 600 samples that have gone through the same high-quality control procedures as previous studies [14, 27, 28], with additional validation using clonal samples that underwent both sequencing and genotyping. Genome annotation and measures of quality control (eg, uniqueness) incorporated into the tool allow a further level of assessment of variant quality. The ultimate validation is likely to come from independent studies using whole-genome sequencing technologies. When these data are placed into the public domain with appropriate meta-data (eg, location of samples) then these raw sequence data will be processed using our proven pipeline and included in *PlasmoView*.

The case studies above demonstrate the utility of *PlasmoView* to support informed decision-making processes, whether they take place in the clinic, laboratory or public policy arena. The genomic variation identified in drug resistance (eg, *PfCRT, PfDHFR* and *PfMDR1*) and vaccine candidate (eg, *PfRH5, MSP3.8* and *AMA1*) genes will help to define the potential repertoire of polymorphisms for follow-up experiments. Similarly, the identification of polymorphisms that drive genetic differentiation at the continental, regional and village level (eg, in drug resistance and *surfin* genes) will facilitate the barcoding of parasites for use in surveillance applications. *PlasmoView* has the functionality to display the variation with annotations, geographical distribution and frequency data and population differentiation metrics in real time.

Whilst *PlasmoView* is presented as a tool to visualise and summarise *Pf* variation, it will be extended to other Plasmodium species as the data becomes available. The next phase of the work is to characterise and display variation other than SNPs, leveraging off the high-coverage, paired-end nature of sequence data [29] and results from genotyping approaches (eg, comparative genomic hybridization [30]). Functionality will expand to include additional population genetic statistics (eg, *Tajima's D* [31]).

In summary, *PlasmoView* is a powerful, scalable tool for the interactive geographic visualisation of *Pf* mutations. Using a high-quality set of polymorphisms, this study shows that *PlasmoView* is useful in confirming existing results, identifying potential avenues for further research and presenting complex genetic data to a broad audience.

## Supplementary Data

Supplementary materials are available at *The Journal of Infectious Diseases* online (http://jid.oxfordjournals.org/). Supplementary materials consist of data provided by the author that are published to benefit the reader. The posted materials are not copyedited. The contents of all supplementary data are the sole responsibility of the authors. Questions or messages regarding errors should be addressed to the author.

Supplementary Data
