# Supplementary material for: PlasmoView: A Web-based Resource to Visualise Global Plasmodium falciparum Genomic Variation
Source: J Infect Dis. 2013 Dec 12;209(11):1808–15. doi: 10.1093/infdis/jit812 (PMC4017360; doi:10.1093/infdis/jit812)
Supplement: Supplementary Data [file supp_209_11_1808__index.html]

PlasmoView: A Web-based Resource to Visualise Global Plasmodium falciparum Genomic Variation — PlasmoView: A Web-based Resource to Visualise Global Plasmodium falciparum Genomic Variation — Supplementary Data 

# *PlasmoView*: A Web-based Resource to Visualise Global *Plasmodium falciparum* Genomic Variation

## Supplementary Data

Supplementary Data

**Files in this Data Supplement:**

- Supplementary Data - Doc file
