# Supplementary material for: PlasmoView: A Web-based Resource to Visualise Global Plasmodium falciparum Genomic Variation
Source: J Infect Dis. 2013 Dec 12;209(11):1808–15. doi: 10.1093/infdis/jit812 (PMC4017360; doi:10.1093/infdis/jit812)

**ONLINE METHODS**

**Data processing**

Publicly-available raw sequence data from *Pf* genomic diversity studies (8,14,21,27,28) were downloaded (32,33). All samples selected underwent paired-end whole-genome sequencing, with a read length at least 54-base pairs and standard fragment sizes, using Illumina® technology (see (14) for a description). The resulting files were processed as described (28). In particular, raw sequence was mapped to the *Pf* 3D7 genome (version 3 (12)) using *smalt* (34) and variants were called using *samtools* (35) and *vcftools* (36) with default settings. Alleles at SNP positions were called using ratios of coverage (see (14)). Samples were removed if they had more than 20% missing genotype calls. Only high-quality SNPs (with <10% missing alleles, <10% mixed calls and minor allele seen at least twice) were included. The final set of ~600k SNPs includes those (86k) determined in a smaller sample set in limited regions of the *Pf* genome (14). The genomic uniqueness at each position was estimated by sliding 54-bp windows across the reference sequence and assessing the frequency of the resulting haplotypes in a blast search of the whole genome. Minor allele frequencies and *F_ST_* statistics (15) are calculated in real-time dependent on the data shown. *PlasmoView* uses the GGV engine (37) with malaria specific additions, data and documentation.

**Supplementary Table 1: Publicly available *P. falciparum* genome-wide sequencing and genotyping datasets included in *PlasmoView***

| **Region** | **Population** | **N*** | **Reference** |
| --- | --- | --- | --- |
| **WAF** | Burkina Faso | 31 | (14,38,39) |
|  | Gambia | 60 | (11,27) |
|  | Ghana | 238 | (27) |
|  | Mali | 32 | (14,38) |
|  | Senegal | 6 | (40) |
| **EAF** | Kenya | 25 | (14,20) |
|  | Malawi | 63 | (21) |
| **SEA** | Cambodia | 71 | (14,21,27) |
|  | Thailand | 68 | (14,21,27,39) |
|  | Vietnam | 12 | (14,27) |
| **OCE** | PNG | 16 | (14,27) |
| **LAB** | Mixed | 8 | (12,14,28,29) |
| **Total** | **11 + 1** | **631** |  |

The parasite genome data used in this study have been made publicly available from the EBI short read archive (accession numbers ERP000190, SRP003502) and PlasmoDB (12). The LAB group includes genotypes of laboratory-adapted parasite lines (from PlasmoDB (12)) and imported parasite isolates (from the London Hospital of Tropical Diseases (28)); * post-quality control filtering.

**Supplementary Figure 1: Analysis of approximately 600,000 single-nucleotide polymorphisms in 624 samples**

**(a) Variant summary and SNP density:** For each of the 14 chromosomes, SNPs are classified by their genomic coding annotation (*Pf* 3D7, version 3.0) as genic (non-synonymous, synonymous and intronic) or intergenic mutations. The overall SNP density is 1 per 39.2 bp (chromosomal range 35.7 bp to 45.2 bp) and shows no significant variation between chromosomes.

**(b) Minor allele frequency (MAF) spectrum**

There is a predominance of low-frequency alleles observed in the 631 samples.

**(c) Rapid decay in linkage disequilibrium (LD)**

The LD between SNPs decreases rapidly with physical distance. Using a 15% MAF threshold, the mean r^2^ LD decreases more rapidly in the African regions (WAF and EAF) than in SEA or OCE. Results are presented as the mean of the r^2^ values in 25-bp bins.

**(d) Population structure**

Using a principal component (PC) analysis based on SNPs with minor allele frequency >15%, there is strong differentiation between the African, SEA and OCE samples. This differentiation is reinforced by 27.9% of SNPs having *F_ST_* >0.1 in between-region comparisons and only 6.6% of SNPs having *F_ST_* >0.1 in intra-region comparisons.

**Supplementary Figure 2: Related screen shots**

**(a) *PfDHFR***

The dihydrofolate reductase gene (*DHFR*, Chromosome 4, *PF3D7_0417200*) contains 8 SNPs, including 2 approaching global fixation and 1 approaching fixation everywhere except OCE. There are two SNPs specific to SEA and OCE, which leads to high *F_ST_* values. The other 3 SNPs comprise rare alleles. This gene is associated with sulfadoxine-pyrimethamine resistance at the four labelled SNPs.

**(b) *PfDHPS***

The dihydropteroate synthase gene (*DHPS*, Chromosome 8, *PF3D7_0821300*) is associated with sulfadoxine-pyrimethamine resistance at the 5 labelled SNPs.

**(c) *PfMDR1***

The multi-drug resistance gene (*MDR1*, Chromosome 5, *PF3D7_0523000*) contains multiple SNPs, 4 of which (labelled) have been associated with drug resistance.

**(d) *Pf* chromosome 13, gene *Pf3D7_1344700***

The view of only one gene from the middle of Figure 2c gives much information: the gene runs from 1791032 to 1794766 (length 3735 bp) with 142 SNPs identified.

**(e) *Pf* chromosome 13, position 1,793,121**

The polymorphism at position 1793121 (SNP 1 in [7]) in *Pf* chromosome 13 has a MAF =17% and an *F_ST_* =0.38 and is highly prevalent in Southeast Asian populations. See Figure 2c and Supplementary Figure 4.

**(f) *MSP3.8***

The MSP3.8 gene (*MSP3.8*, Chromosome 10, *PF3D7_1036300*) has been found to be under balancing selection in a number of studies, including in parasite isolates from The Gambia, Kenya and Malawi. The high number of SNPs with intermediate MAF and low *F_ST_* reinforce these conclusions, but on a global scale in *PlasmoView*.


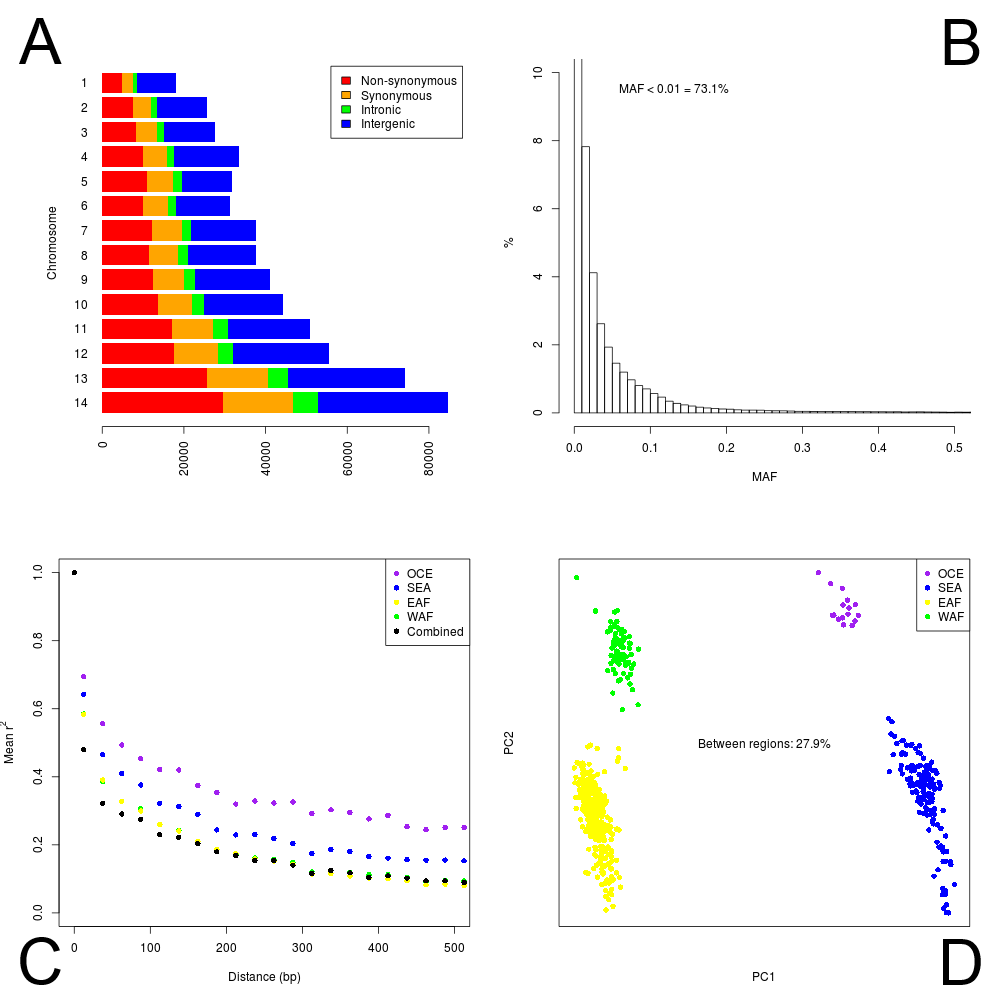


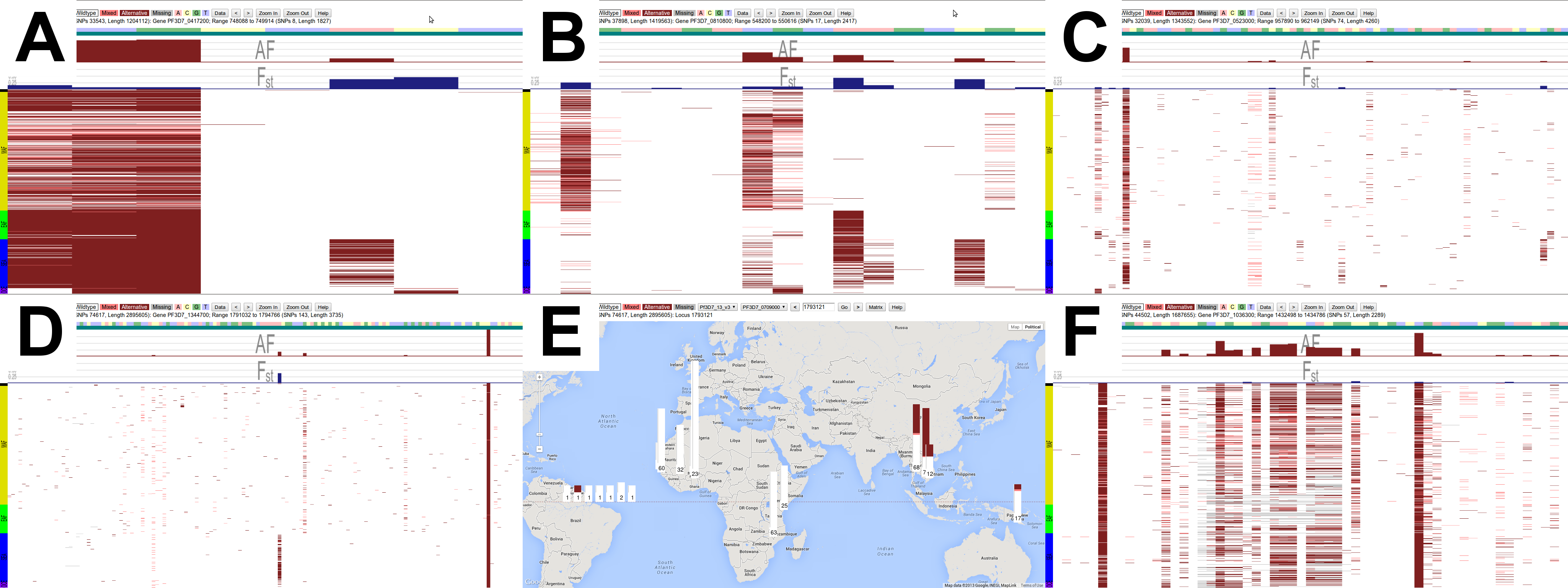

Supplement: Supplementary Data [file supp_jit812_jit812supp.doc]
